# Reproducibility and Validity of Dietary Patterns Assessed by a Food Frequency Questionnaire Used in the 5-Year Follow-Up Survey of the Japan Public Health Center-Based Prospective Study

**DOI:** 10.2188/jea.JE20110087

**Published:** 2012-05-05

**Authors:** Akiko Nanri, Taichi Shimazu, Junko Ishihara, Ribeka Takachi, Tetsuya Mizoue, Manami Inoue, Shoichiro Tsugane

**Affiliations:** 1 Department of Epidemiology and Prevention, International Clinical Research Center, National Center for Global Health and Medicine, Tokyo, Japan; 2 Epidemiology and Prevention Division, Research Center for Cancer Prevention and Screening, National Cancer Center, Tokyo, Japan; 3 Department of Nutrition, Junior College of Tokyo University of Agriculture, Tokyo, Japan; 4 Department of Community Preventive Medicine, Division of Social and Environmental Medicine, Niigata University Graduate School of Medical and Dental Sciences, Niigata, Japan

**Keywords:** dietary patterns, Japanese, reproducibility, validity

## Abstract

**Background:**

Analysis of dietary pattern is increasingly popular in nutritional epidemiology. However, few studies have examined the validity and reproducibility of dietary patterns. We assessed the reproducibility and validity of dietary patterns identified by a food frequency questionnaire (FFQ) used in the 5-year follow-up survey of the Japan Public Health Center-Based Prospective Study (JPHC Study).

**Methods:**

The participants were a subsample (244 men and 254 women) from the JPHC Study. Principal component analysis was used to identify dietary patterns from 28- or 14-day dietary records and 2 FFQs. To assess reproducibility and validity, we calculated Spearman correlation coefficients between dietary pattern scores derived from FFQs separated by a 1-year interval, and between dietary pattern scores derived from dietary records and those derived from a FFQ completed after the dietary records, respectively.

**Results:**

We identified 3 Japanese dietary patterns from the dietary records and 2 FFQs: prudent, westernized, and traditional. Regarding reproducibility, Spearman correlation coefficients between the 2 FFQs ranged from 0.55 for the westernized Japanese pattern in men and the prudent Japanese pattern in women to 0.77 for the traditional Japanese pattern in men. Regarding validity, the corresponding values between dietary records and the FFQ ranged from 0.32 for the westernized Japanese pattern in men to 0.63 for the traditional Japanese pattern in women.

**Conclusions:**

Acceptable reproducibility and validity was shown by the 3 dietary patterns identified by principal component analysis based on the FFQ used in the 5-year follow-up survey of the JPHC Study.

## INTRODUCTION

In nutritional epidemiology, there is increased interest in the analysis of dietary pattern, a comprehensive variable that integrates consumption of several foods or food groups. The effect of a single nutrient, food, or food group on disease risk and morbid conditions is difficult to assess in observational studies because foods and nutrients are consumed in combination and their complex effects are likely to be interactive or synergistic.^1^ However, dietary pattern can overcome problems relating to the close intercorrelation among foods or nutrients and is expected to have a greater impact on disease risk than any single nutrient.^1^^–^^3^ Therefore, analysis using dietary pattern could be used as a complementary approach in nutritional epidemiology. Indeed, many previous studies have reported associations of dietary patterns with mortality, several diseases (such as cancer, cardiovascular disease, and type 2 diabetes), and biomarkers.^4^^,^^5^

Extraction of dietary pattern by principal component analysis—which is often used to identify dietary patterns—requires some arbitrary decisions to group food items, determine the number of factors to retain, select the method of rotation of the initial factors, and label the dietary patterns.^6^ Moreover, dietary patterns may differ with regard not only to age and sex but also resident area, ethnic group, and culture. Therefore, it might be necessary to examine the validity and reproducibility of the identified dietary patterns among the study population of each study. To date, several studies have examined the reproducibility and validity of dietary patterns.^7^^–^^13^ However, among studies of the association between dietary patterns and disease risk in Japan, only 1 examined the validity of dietary patterns in Japan^12^ and none examined reproducibility. Therefore, we assessed the reproducibility and validity of dietary patterns identified by principal component analysis of a food frequency questionnaire (FFQ) used in the 5-year follow-up survey of the Japan Public Health Center-Based Prospective Study (JPHC Study).

## METHODS

### JPHC Study

The JPHC Study is a population-based prospective study of cancer and cardiovascular disease and was launched in 1990 for cohort I and in 1993 for cohort II.^14^ The participants were 140 420 residents of 11 public health center areas and were aged 40 to 59 years for cohort I and aged 40 to 69 years for cohort II at each baseline survey. At baseline and at the 5- and 10-year follow-ups, a questionnaire survey was conducted to obtain information on medical histories and health-related lifestyle, including diet.

### Study population

The subjects of the reproducibility and validity study of the FFQ used in the 5-year follow-up survey were a subsample of the participants in JPHC Study cohorts I and II. Married couples were recruited. The present study used a data set for 498 subjects (244 men and 254 women; 209 in cohort I and 289 in cohort II) who completed 2 FFQs with a 1-year interval and dietary records for a total of 28 or 14 days. Details of the validation study have been described elsewhere.^15^^,^^16^ There was no significant difference in mean age between participants of the validation study and cohort members among men and women in cohort I or among men in cohort II; however, there was a 3-year age difference among cohort II women (mean age, 56 years for the validation subsample vs 59 years for the cohort participants). In the present study, we identified dietary patterns using FFQ data from the 5-year follow-up survey. Oral or written informed consent from the participants was received before the study. The study did not undergo ethical approval since it was conducted before the advent of ethical guidelines for epidemiology research in Japan, which mandate such approval.

### Dietary assessment

The participants completed the FFQ twice, with an approximately 1-year interval. The FFQ for the evaluation of validity (FFQ_V) was completed after completion of the last dietary record and was compared with the dietary records. The FFQ for the evaluation of reproducibility (FFQ_R) was administered 1 year before or after the FFQ_V and was compared with the FFQ_V. The FFQ included questions on 138 food items (with standard portions/units and eating frequency) and 14 supplementary questions during the previous year, from which a composition table was developed for 147 foods items.^17^ For most food items, 9 response options were available for eating frequency: rarely, 1 to 3 times/month, 1 to 2 times/week, 3 to 4 times/week, 5 to 6 times/week, once a day, 2 to 3 times/day, 4 to 6 times/day, and 7 or more times/day. Slightly different options were used for beverage intake: rarely, 1 to 2 times/week, 3 to 4 times/week, 5 to 6 times/week, 1 cup/day, 2 to 3 cups/day, 4 to 6 cups/day, 7 to 9 cups/day, and 10 or more cups/day. A standard portion size was specified for each food item, and the respondents were asked to choose their usual portion size from 3 options (less than half the standard portion size, standard portion size, or more than 1.5 times the standard portion size). We calculated daily intake of most foods by multiplying daily frequency of consumption by usual portion size. In the present analysis, we used 134 food and beverage items of the FFQ (excluding 11 items that correlated strongly with others and 2 items with no energy or nutrition).

The participants completed a 28- or 14-day (Okinawa) dietary record in 1 year, ie, 7-day dietary records were collected 4 (or 2) times at 3-month (or 6-month) intervals during the course of a year. The survey method using dietary records has been described elsewhere.^15^^,^^16^^,^^18^ We matched the 1101 unique food codes in the dietary records to food items on the FFQ to ensure that daily food intakes derived from the dietary records were comparable with those derived from the FFQ. A total of 558 food codes in the dietary records were matched to the 134 food items on the FFQ.

### Statistical analysis

Men and women were analyzed separately. Some foods or food groups that were similar in nutritional content or culinary use were combined, leaving 48 food groups for the present analysis (Table 1). When information from dietary records was used to combine 2 or more foods into 1 food group, the amount of food before cooking was used for all food items except noodles and rice. Consumption of noodles and rice was expressed in weight of boiled noodles and steamed rice, respectively. Consumption of miso soup and beverages such as coffee and green tea was expressed as the weight of the consumed infusion rather than the weight of miso, coffee beans, instant coffee, or tea leaves. We calculated the means and standard deviations of each food group intake estimated from dietary records and the 2 FFQs. In addition, the reproducibility and validity of 48 food group intakes were evaluated by using Spearman correlation coefficients between intakes from the 2 FFQs and between intakes derived from the dietary records and those derived from the FFQ_V.

**Table 1.** Food groupings used in the dietary pattern analyses

Foods or food groups	Food items
Noodle	Buckwheat noodles, Japanese wheat noodles, Chinese noodles, Okinawa noodles
Rice	Rice, rice cake
Bread	Bread
Potatoes	Potatoes, sweet potatoes, taros
Soy products	Tofu, yushi-dofu, koya-dofu, aburaage, natto, soy milk
Miso soup	Miso soup
Peanuts	Peanuts
Green, leafy vegetables	Spinach, Chinese chive, garland chrysanthemum, komatsuna, qing gin cai, leaf mustard, Swiss chard, mugwort
Other green vegetables	Green pepper, kidney beans, broccoli
Dark-yellow vegetables	Carrots, pumpkin
Tomatoes	Tomatoes, tomato juice
Other vegetables	Cabbage, Japanese radish, onion, cucumber, Chinese cabbage, bean sprout, lettuce, bitter gourd, sponge gourd
Pickles	Takuan-zuke, pickled green leaf, pickled plum, pickled Chinese cabbage, pickled cucumber, pickled eggplant
Other fruit	Papayas, apples, Japanese persimmons, strawberries, grapes, melon, watermelon, peaches, pears, kiwifruit, pineapples, bananas
Citrus fruit	Mandarin orange, citrus fruit
Fruit juice	Orange juice, apple juice
Mushroom	Shiitake, enokidake mushroom
Seaweed	Wakame, hijiki, laver
Seafood other than fish	Squids, octopuses, shrimps, clam, pond snail
Oily fish	Horse mackerel/sardine, pacific saury/mackerel, sea bream
Salmon	Salmon
Eel	Eel
Lean fish	Cod/flatfish, skipjack/tuna
Salty fish	Salted fillet, dried fish, salted cod roe, dried whitebait
Fish products	Canned tuna, fish-paste products
Pork	Stir-fried pork, deep-fried pork, stewed pork
Beef	Steak, broiled beef, stewed beef (curry/stew)
Chicken	Broiled chicken, deep-fried chicken
Liver	Liver
Processed meats	Ham, sausage, bacon, luncheon meat
Egg	Egg
Milk	Milk
Dairy products	Yogurt, cheese, Yakult (lactic-acid bacteria beverages)
Soup	Soup
Confectioneries	Japanese confectionaries, cake, biscuit, chocolate
Green tea	Green tea
Coffee	Coffee, canned coffee
Soft drink	Cola drink, energy drinks
Oolong tea	Oolong tea
Black tea	Black tea
Sauce	Sauce, ketchup
Mayonnaise	Mayonnaise
Dressing	Dressing
Sake	Sake
Shochu	Shochu
Beer	Beer
Whisky	Whisky
Wine	Wine

To identify dietary patterns, we performed principal component analysis based on log-transformed intakes of 48 food groups for each of the 2 FFQs and the dietary records. The factors were rotated by orthogonal transformation (varimax rotation) to maintain uncorrelated factors and greater interpretability. We considered eigenvalues, the scree plot, and the interpretability of the factors to determine the number of factors to retain and identified 3 dietary patterns (factors) in both men and women. The dietary patterns were named according to the food items showing high loading (absolute value) in each dietary pattern. The factor scores for each dietary pattern were calculated by summing intakes of food items weighted by their factor loadings. The scores were energy-adjusted using the residual method. When we examined reproducibility and validity by using dietary pattern scores without energy adjustment, or based on energy-adjusted intake of food groups, the derived dietary patterns and their reproducibility and validity were similar to those based on energy-adjusted scores.

We examined the correspondence of each dietary pattern identified from the dietary records with dietary patterns extracted from the FFQ_V by comparing the pattern of food items showing high loading. We repeated this procedure to link dietary patterns identified from the FFQ_R and FFQ_V. For each dietary pattern, Spearman correlation coefficients between the factor score for the dietary patterns derived from the dietary records and those derived from the FFQ_V were calculated to evaluate the validity of the FFQ. In addition, to determine the reproducibility of the FFQ, Spearman correlation coefficients between the factor scores for the dietary patterns derived from the 2 FFQs administered 1 year apart were calculated. We estimated Spearman correlation coefficients between factor loadings from the FFQ in each population (subsample of the validity study, overall cohort, men, women, and men and women combined) to compare the dietary patterns derived from the FFQ between the subsample of the validity study and overall cohort and between men or women and men and women combined in the overall cohort. All analyses were performed using Statistical Analysis System (SAS) version 9.1 (SAS Institute, Cary, NC, USA).

## RESULTS

The 48 food group intakes calculated by 28- or 14-day dietary records and the 2 FFQs, and their correlations, are shown in Table 2 for men and Table 3 for women. Regarding reproducibility, Spearman correlation coefficients between the 2 FFQs ranged from 0.21 for wine to 0.80 for coffee in men (Table 2) and from 0.37 for *sake* to 0.81 for miso soup in women (Table 3). In particular, intakes of rice, bread, miso soup, pickles, other fruit, salt fish, processed meat, milk, dairy products, green tea, and coffee showed high correlation coefficients (*r* >0.60) between the 2 FFQs in both men and women. Regarding validity, Spearman correlation coefficients between the dietary records and the FFQ_V ranged from 0.06 for oily fish to 0.75 for coffee in men (Table 2) and from 0.12 for *shochu* to 0.80 for coffee in women (Table 3). In both men and women, intakes of coffee, bread, pickles, and milk estimated by the FFQ_V showed a high correlation (*r* >0.60) with those estimated by the dietary records, whereas the validity of oily fish between the dietary records and the FFQ_V was very low.

**Table 2.** Food group intakes (g/day) calculated from 28- or 14-day DRs and 2 FFQs and their correlations in men (*n* = 244)

Food groups	FFQ_R^a^	FFQ_V^b^	DR^c^	Spearman correlations	
			
	Mean (SD)	Mean (SD)	Mean (SD)	FFQ_R vs FFQ_V	DR vs FFQ_V
Noodle	119.9 (146.8)	111.3 (93.0)	62.6 (43.3)	0.49	0.44
Rice	438.7 (200.5)	417.0 (202.1)	479.9 (159.0)	0.79	0.67
Bread	24.3 (52.4)	18.9 (25.3)	20.7 (23.4)	0.70	0.65
Potatoes	30.4 (28.1)	28.0 (27.5)	34.4 (19.4)	0.54	0.21
Soy products	67.4 (52.3)	70.3 (53.2)	68.3 (32.0)	0.62	0.39
Miso soup	240.0 (162.0)	227.8 (173.7)	204.4 (100.6)	0.79	0.51
Nuts	2.6 (4.3)	2.3 (3.8)	1.0 (2.5)	0.56	0.25
Green, leafy vegetables	32.7 (35.2)	33.7 (36.2)	24.8 (16.0)	0.52	0.33
Other green vegetables	13.6 (15.1)	13.2 (14.3)	11.9 (8.5)	0.61	0.26
Dark-yellow vegetables	30.3 (32.0)	29.9 (28.4)	31.1 (15.3)	0.51	0.45
Tomatoes	33.9 (43.4)	32.6 (42.2)	25.4 (28.5)	0.55	0.42
Other vegetables	109.0 (80.1)	108.1 (75.4)	134.6 (44.7)	0.59	0.25
Pickles	42.4 (53.2)	38.7 (49.4)	23.1 (24.2)	0.76	0.72
Other fruit	121.3 (127.3)	116.1 (100.2)	78.0 (58.8)	0.68	0.51
Citrus fruit	67.4 (81.4)	75.4 (85.0)	25.1 (23.0)	0.60	0.48
Fruit juice	40.4 (62.2)	35.8 (59.9)	3.7 (11.5)	0.52	0.22
Mushroom	11.5 (11.6)	11.4 (9.8)	11.2 (7.2)	0.54	0.37
Seaweed	12.4 (14.5)	11.9 (10.2)	7.8 (6.7)	0.50	0.24
Seafood other than fish	17.5 (14.5)	17.1 (14.6)	20.9 (12.1)	0.50	0.37
Oily fish	28.5 (25.1)	26.3 (22.2)	23.6 (17.9)	0.51	0.06
Salmon	10.0 (13.0)	8.3 (10.4)	10.3 (10.3)	0.59	0.51
Eel	2.3 (2.8)	2.3 (2.6)	2.0 (2.9)	0.59	0.25
Lean fish	14.5 (13.3)	13.5 (10.9)	21.9 (15.1)	0.58	0.33
Salty fish	21.8 (26.6)	23.8 (40.2)	11.5 (9.7)	0.73	0.57
Fish products	7.9 (7.1)	7.4 (6.6)	8.3 (5.5)	0.55	0.20
Pork	32.3 (30.7)	29.7 (25.0)	28.2 (15.1)	0.49	0.42
Beef	16.0 (13.9)	16.3 (16.0)	15.3 (12.2)	0.55	0.43
Chicken	10.5 (11.9)	10.6 (12.5)	16.0 (12.1)	0.52	0.20
Liver	2.9 (3.8)	2.7 (3.8)	1.4 (3.0)	0.58	0.30
Processed meats	7.3 (7.8)	7.3 (9.2)	9.0 (8.1)	0.60	0.45
Eggs	30.8 (21.7)	31.8 (28.2)	38.3 (14.8)	0.56	0.51
Milk	179.4 (232.8)	163.9 (179.0)	101.6 (87.9)	0.66	0.69
Dairy products	35.9 (67.3)	33.3 (45.2)	17.5 (35.2)	0.72	0.67
Soup	1.9 (3.7)	1.7 (3.5)	—	0.36	—
Confectionaries	18.6 (21.1)	17.8 (20.6)	21.7 (19.1)	0.71	0.51
Green tea	609.7 (510.5)	559.1 (421.1)	301.4 (235.5)	0.61	0.44
Coffee	148.8 (194.1)	125.9 (155.2)	81.8 (116.5)	0.80	0.75
Soft drink	62.7 (104.0)	62.9 (126.9)	24.9 (56.7)	0.61	0.35
Oolong tea	41.5 (128.5)	35.8 (89.7)	32.3 (65.8)	0.57	0.26
Black tea	18.5 (88.2)	12.8 (28.1)	6.3 (20.3)	0.56	0.45
Sauce	1.3 (1.5)	1.3 (1.5)	2.5 (2.1)	0.52	0.23
Mayonnaise	1.3 (1.6)	1.2 (1.4)	2.1 (1.7)	0.40	0.42
Dressing	1.7 (2.6)	1.4 (2.0)	0.8 (1.3)	0.53	0.32
Sake	76.1 (128.4)	80.0 (137.6)	59.7 (94.2)	0.78	0.72
Shochu	44.0 (116.9)	47.1 (108.9)	30.2 (84.4)	0.76	0.67
Beer	147.5 (245.5)	143.2 (261.4)	196.4 (223.0)	0.58	0.53
Whisky	3.6 (15.2)	3.6 (16.1)	4.4 (16.5)	0.36	0.37
Wine	2.3 (13.1)	3.7 (19.6)	3.2 (18.0)	0.21	0.13

Abbreviations: DR, dietary record; FFQ, food frequency questionnaire; SD, standard deviation.

^a^FFQ_R was administered 1 year after or before FFQ_V.

^b^FFQ_V was administered after completion of the DRs.

^c^28- and 14-day DRs were collected over a 1-year period.

**Table 3.** Food group intakes (g/day) calculated from 28- or 14-day DRs and 2 FFQs and their correlations in women (*n* = 254)

Food groups	FFQ_R^a^	FFQ_V^b^	DR^c^	Spearman correlations	
			
	Mean (SD)	Mean (SD)	Mean (SD)	FFQ_R vs FFQ_V	DR vs FFQ_V
Noodle	83.5 (67.2)	88.8 (73.0)	43.8 (27.3)	0.57	0.43
Rice	333.7 (146.4)	327.8 (141.1)	315.3 (100.9)	0.71	0.54
Bread	28.8 (40.9)	26.3 (32.1)	25.0 (22.3)	0.67	0.63
Potatoes	35.9 (33.6)	35.3 (29.4)	34.9 (17.4)	0.61	0.30
Soy products	71.5 (62.9)	73.3 (51.6)	58.0 (24.1)	0.56	0.33
Miso soup	206.2 (135.0)	192.9 (135.9)	171.6 (82.5)	0.81	0.51
Nuts	2.2 (5.5)	2.2 (3.5)	0.6 (1.5)	0.56	0.19
Green, leafy vegetables	39.2 (38.7)	39.6 (40.8)	23.2 (14.5)	0.58	0.31
Other green vegetables	18.2 (21.4)	16.2 (15.6)	11.3 (7.4)	0.57	0.31
Dark-yellow vegetables	35.4 (32.2)	37.8 (35.6)	30.9 (14.4)	0.56	0.45
Tomatoes	36.0 (44.1)	32.5 (33.0)	29.2 (25.0)	0.58	0.43
Other vegetables	121.5 (97.9)	117.3 (77.7)	118.5 (37.5)	0.58	0.22
Pickles	41.6 (51.4)	38.5 (45.6)	19.0 (18.8)	0.74	0.76
Other fruit	166.4 (211.7)	145.1 (134.6)	97.8 (57.0)	0.66	0.41
Citrus fruit	99.1 (123.0)	98.5 (95.3)	37.5 (27.4)	0.57	0.36
Fruit juice	38.5 (54.2)	39.8 (61.5)	5.1 (10.7)	0.51	0.31
Mushroom	13.2 (10.6)	13.3 (11.1)	10.5 (6.3)	0.53	0.34
Seaweed	13.3 (12.1)	12.3 (9.8)	7.1 (5.2)	0.54	0.18
Seafood other than fish	16.1 (14.0)	14.5 (12.5)	15.2 (8.6)	0.57	0.29
Oily fish	27.4 (31.0)	23.2 (18.3)	16.6 (10.6)	0.52	0.14
Salmon	9.7 (18.4)	8.1 (10.3)	8.7 (8.2)	0.58	0.49
Eel	1.9 (2.2)	1.7 (2.0)	1.3 (2.1)	0.67	0.35
Lean fish	12.7 (10.8)	12.2 (11.2)	14.4 (9.4)	0.52	0.34
Salty fish	20.6 (24.0)	19.5 (21.0)	9.3 (7.2)	0.69	0.47
Fish products	9.2 (9.1)	7.8 (6.7)	7.3 (4.9)	0.66	0.25
Pork	28.7 (25.2)	25.1 (27.3)	21.2 (11.9)	0.54	0.38
Beef	11.6 (12.2)	11.8 (12.6)	10.0 (8.5)	0.61	0.53
Chicken	9.2 (9.3)	8.7 (10.5)	13.7 (11.8)	0.53	0.27
Liver	2.6 (4.2)	2.4 (4.0)	1.1 (2.5)	0.63	0.35
Processed meats	7.2 (7.0)	6.8 (7.8)	7.4 (5.5)	0.62	0.35
Eggs	29.4 (19.0)	29.3 (26.7)	31.4 (12.6)	0.65	0.53
Milk	195.0 (216.7)	181.0 (208.2)	126.7 (86.3)	0.75	0.72
Dairy products	47.9 (49.0)	50.9 (61.9)	26.3 (32.6)	0.67	0.58
Soup	2.3 (3.9)	1.9 (3.5)	—	0.46	—
Confectionaries	26.5 (29.6)	25.7 (23.9)	35.1 (22.7)	0.62	0.43
Green tea	604.9 (503.7)	576.7 (437.6)	302.6 (222.5)	0.70	0.53
Coffee	109.0 (131.3)	112.2 (144.5)	76.2 (90.4)	0.80	0.80
Soft drink	42.9 (68.6)	48.9 (160.8)	17.4 (29.8)	0.51	0.41
Oolong tea	44.8 (117.9)	40.2 (87.4)	35.0 (59.5)	0.55	0.38
Black tea	19.6 (45.5)	23.9 (56.5)	12.3 (41.5)	0.56	0.54
Sauce	1.4 (1.5)	1.3 (1.4)	2.2 (1.7)	0.50	0.26
Mayonnaise	1.4 (1.8)	1.2 (1.3)	2.2 (1.7)	0.47	0.46
Dressing	1.9 (2.4)	1.8 (2.4)	0.9 (1.4)	0.52	0.32
Sake	2.5 (23.1)	2.0 (12.6)	5.0 (13.2)	0.37	0.25
Shochu	1.9 (23.3)	0.0 (0.2)	0.9 (3.2)	0.58	0.12
Beer	18.4 (79.6)	10.6 (45.8)	23.3 (57.2)	0.54	0.54
Whisky	0.0 (0.5)	0.0 (0.4)	0.3 (2.7)	0.41	0.31
Wine	0.7 (4.8)	0.7 (5.0)	1.1 (5.6)	0.48	0.33

Abbreviations: DR, dietary record; FFQ, food frequency questionnaire; SD, standard deviation.

^a^FFQ_R was administered 1 year after or before FFQ_V.

^b^FFQ_V was administered after completion of the DRs.

^c^28- and 14-day DRs were collected over a 1-year period.

Principal component analysis identified 3 dietary patterns from the dietary records and 2 FFQs (Table 4 for men and Table 5 for women), and they were similar in terms of factor loading pattern across the data sources and between sexes. Of the 3 dietary patterns, a pattern highly loaded by intakes of vegetables, fruit, potatoes, soy products, mushrooms, seaweed, oily fish, and green tea was named the prudent Japanese pattern. A dietary pattern associated with high intakes of bread, meat, processed meat, fruit juice, coffee, black tea, soft drinks, sauces, mayonnaise, and dressing was named the westernized Japanese pattern. Another dietary pattern was characterized by high intakes of rice, miso soup, pickles, salmon, salty fish, seafood other than fish, fruit, and *sake* (men only), and was named the traditional Japanese pattern. The 3 dietary patterns from the dietary records, the FFQ_R, and the FFQ_V explained 23.9%, 29.4%, and 26.5%, respectively, of variance in men and 23.0%, 24.9%, and 32.9%, respectively, of variance in women.

**Table 4.** Factor-loading matrix for 3 Japanese dietary patterns identified by principal component analysis^a^ in men (*n* = 244)

	Prudent			Westernized			Traditional		
			
	FFQ_R^b^	FFQ_V^c^	DR^d^	FFQ_R	FFQ_V	DR	FFQ_R	FFQ_V	DR
Noodle	0.35		−0.19	0.29	0.39	0.15	0.22	−0.12	0.47
Rice			0.12			−0.54	0.34	0.30	0.19
Bread	0.19			0.31	0.18	0.46	−0.23	−0.30	
Potatoes	0.63	0.50	0.41		0.36		0.11		
Soy products	0.54	0.38	0.42		0.33	−0.24			
Miso soup	0.23					−0.65	0.25	0.29	0.20
Nuts	0.29	0.27	0.11	0.36	0.44		0.26		0.33
Green, leafy vegetables	0.67	0.78	0.45				−0.17	−0.10	
Other green vegetables	0.74	0.67	0.40	0.13		0.12	0.12	0.17	
Dark-yellow vegetables	0.66	0.72	0.71	−0.15	−0.13	0.13	−0.18		−0.19
Tomatoes	0.55	0.53	0.37		0.12	0.19			0.52
Other vegetables	0.65	0.75	0.70	0.21		−0.13	0.13		−0.19
Pickles	0.25	0.17			0.29	−0.26	0.53	0.61	0.59
Other fruit	0.52	0.63	0.14			0.14	0.32	0.29	0.62
Citrus fruit	0.28	0.46	0.29	0.11			0.25	0.39	0.48
Fruit juice	0.13			0.35	0.44	0.26		−0.11	0.18
Mushroom	0.64	0.36	0.38	0.21	0.52		0.19		0.28
Seaweed	0.75	0.57	0.39		0.31				0.13
Seafood other than fish	0.61			0.30	0.64	−0.12	0.21	0.19	0.39
Oily fish	0.67	0.32	0.26	0.17	0.44	−0.15	0.13		−0.22
Salmon	0.19	0.13		0.18	0.40	−0.23	0.57	0.42	0.72
Eel	0.30		0.10	0.23	0.42	0.46	−0.25	−0.30	0.13
Lean fish	0.37	0.20	0.14	0.26	0.29	−0.26	0.26		
Salty fish	0.37	0.10			0.39	−0.13	0.62	0.60	0.74
Fish products	0.44	0.29	0.25	0.29	0.11	0.12		−0.26	−0.23
Pork	0.10		0.24	0.62	0.45				−0.41
Beef		−0.11		0.53	0.45	0.36	−0.22	−0.34	−0.24
Chicken		0.17		0.68	0.33	0.30	0.22		
Liver	0.17		0.21	0.36	0.24				−0.23
Processed meats	0.16	0.20		0.56	0.29	0.27		−0.33	−0.21
Eggs	0.16	0.33	0.23	0.18					0.11
Milk	0.29	0.26	0.29						0.41
Dairy products	0.35	0.30	0.19	0.20	0.18	0.31		−0.14	0.30
Soup	0.13			0.34	0.33		−0.47	−0.42	
Confectionaries	0.34	0.30		0.30	0.21	0.19	0.16		0.43
Green tea	0.49	0.14	0.24		0.45	0.11	0.35	0.25	0.39
Coffee				0.35	0.14	0.36	−0.17	−0.35	−0.11
Soft drink	−0.10		−0.16	0.49	0.38	0.17		−0.23	0.10
Oolong tea				0.33	0.27	0.29	−0.32	−0.45	
Black tea		0.18		0.24	0.30	0.47	−0.15	−0.25	0.17
Sauce	0.33	0.13		0.42	0.50	0.41			0.13
Mayonnaise	0.34	0.21		0.32	0.39	0.14	0.16		0.50
Dressing	0.37	0.31	0.31	0.29	0.27	0.26	−0.19	−0.21	0.12
Sake			−0.11	−0.13	0.12	−0.18	0.63	0.55	0.63
Shochu		−0.12	0.19	0.11		0.30	−0.45	−0.44	−0.27
Beer		−0.12		0.22			−0.19	−0.19	0.17
Whisky	−0.16	−0.25	−0.19	0.27	0.20	0.20			
Wine					0.14	0.45	0.12		0.18

Abbreviations: FFQ, food frequency questionnaire; DR, dietary record.

^a^For simplicity, factor loadings less than ±0.10 are not listed.

^b^FFQ_R was administered 1 year after or before FFQ_V.

^c^FFQ_V was administered after completion of the DRs.

^d^28- and 14-day DRs were collected over a 1-year period.

**Table 5.** Factor-loading matrix for 3 Japanese dietary patterns identified by principal component analysis^a^ in women (*n* = 254)

	Prudent			Westernized			Traditional		
			
	FFQ_R^b^	FFQ_V^c^	DR^d^	FFQ_R	FFQ_V	DR	FFQ_R	FFQ_V	DR
Noodle	0.12	0.54		0.23	0.30		0.41	0.27	0.39
Rice	−0.12				−0.14	−0.50	0.14	0.13	0.23
Bread		0.17	−0.14	0.41	0.42	0.50			−0.13
Potatoes	0.64	0.65	0.48	0.13	0.10	−0.16	0.14	0.33	−0.16
Soy products	0.43	0.77	0.41	0.22	0.16	−0.21	0.12		0.12
Miso soup		0.53				−0.45	0.21	0.19	0.35
Nuts	0.13	0.18	0.22	0.31	0.25	0.17	0.39	0.41	0.19
Green, leafy vegetables	0.67	0.85	0.38	0.18					0.16
Other green vegetables	0.63	0.75	0.46		−0.11	0.19	0.29	0.30	
Dark-yellow vegetables	0.61	0.82	0.63			−0.14	−0.20		−0.35
Tomatoes	0.31	0.45	0.45				0.34	0.27	0.39
Other vegetables	0.76	0.90	0.54						−0.23
Pickles	0.26	0.36		−0.14	−0.15	−0.22	0.64	0.72	0.71
Other fruit	0.62	0.76	0.38			0.16	0.43	0.35	0.53
Citrus fruit	0.42	0.57	0.57		−0.17		0.44	0.30	0.15
Fruit juice				0.39	0.43	0.16	0.25	0.29	0.22
Mushroom	0.43	0.62	0.53				0.16	0.32	
Seaweed	0.63	0.67	0.33	0.15					
Seafood other than fish	0.27	0.40		0.18	0.20		0.48	0.45	0.42
Oily fish	0.50	0.52	0.11	0.18	0.28		0.20		−0.13
Salmon	0.14	0.24		−0.13		−0.16	0.58	0.39	0.59
Eel	0.21		0.29	0.38	0.53	0.33		0.11	
Lean fish	0.15	0.41		0.12	0.12		0.18	0.12	0.20
Salty fish	0.14	0.19	0.30	−0.15	−0.12	−0.14	0.73	0.74	0.71
Fish products	0.17	0.35	0.13	0.39	0.28	0.13	−0.16	−0.11	−0.26
Pork	0.21	0.32		0.48	0.32	0.16	0.12	0.14	−0.25
Beef				0.46	0.50	0.49		0.12	
Chicken		0.17		0.35	0.26	0.32	0.42	0.45	−0.13
Liver	0.12	0.11	0.24	0.34	0.32	0.20		0.15	−0.19
Processed meats		0.22		0.57	0.43	0.33	0.11		
Eggs		0.40	0.18	0.41	0.27	0.25	0.12		0.11
Milk		0.23	0.42	0.27	0.24	0.20			
Dairy products	0.22	0.23	0.37		0.40	0.29	0.33	0.19	0.22
Soup				0.50	0.55		−0.14		
Confectionaries	0.13	0.35		0.50	0.26	0.16	0.20	0.35	0.38
Green tea	0.29	0.40	0.47		−0.22		0.10	0.30	0.34
Coffee	−0.18		−0.23	0.41	0.41	0.42	−0.10		−0.16
Soft drink			−0.14	0.30	0.41	0.25	0.14	0.16	0.22
Oolong tea	−0.21		−0.14	0.30	0.38	0.30		0.13	
Black tea				0.36	0.49	0.42			0.13
Sauce	0.17	0.18		0.27	0.30	0.45	0.38	0.50	
Mayonnaise		0.17	0.14	0.32	0.20	0.15	0.40	0.63	0.48
Dressing		0.15	0.19	0.36	0.32	0.33	0.22	0.32	
Sake		−0.10	0.28				0.19	0.21	0.51
Shochu	−0.16			−0.14	0.16	0.26			
Beer	−0.12				0.23	0.24	0.24	0.23	0.33
Whisky						0.24	0.16		0.17
Wine	−0.12	−0.14			0.28	0.42			0.25

Abbreviations: FFQ, food frequency questionnaire; DR, dietary record.

^a^For simplicity, factor loadings less than ±0.10 are not listed.

^b^FFQ_R was administered 1 year after or before FFQ_V.

^c^FFQ_V was administered after completion of the DRs.

^d^28- and 14-day DRs were collected over a 1-year period.

Three similar dietary patterns were identified in the overall cohort for both men and women (Appendix). The Spearman correlation coefficients between factor loadings from FFQ_R in the subsample of the validity study and those from the FFQ in the overall cohort for 48 food groups were 0.89 in men and 0.82 in women for the prudent Japanese pattern, 0.82 in men and 0.75 in women for the westernized Japanese pattern, and 0.75 in men and 0.47 in women for the traditional Japanese pattern. In the overall cohort, the corresponding values between factor loadings for men or women and those for men and women combined were 0.98 in both men and women for the prudent Japanese pattern, 0.96 in men and 0.95 in women for the westernized Japanese pattern, and 0.76 in men and 0.74 in women for the traditional Japanese pattern.

The Spearman correlation coefficients between the dietary records and the 2 FFQs are shown in Table 6. Regarding reproducibility between the 2 FFQs, all 3 dietary patterns were acceptable in both men and women. In particular, the traditional Japanese pattern in men and the westernized Japanese pattern in women were highly reproduced (correlation coefficients: 0.77 in men and 0.71 in women). Regarding validity, the traditional Japanese pattern had higher correlation coefficients between the dietary records and the FFQ_V than did other patterns among both men and women (correlation coefficient: 0.49 in men and 0.63 in women), whereas the westernized Japanese pattern in men (0.32) had the lowest correlation coefficient among the 3 dietary patterns.

**Table 6.** Spearman correlation coefficients for the prudent, westernized, and traditional dietary pattern scores^a^ between the DRs and the 2 FFQs

	Prudent	Westernized	Traditional
Men			
FFQ_R^b^ vs FFQ_V^c^	0.56	0.55	0.77
DR^d^ vs FFQ_V	0.47	0.32	0.49

Women			
FFQ_R^b^ vs FFQ_V^c^	0.55	0.71	0.68
DR^d^ vs FFQ_V	0.36	0.56	0.63

Abbreviations: DR, dietary record; FFQ, food frequency questionnaire.

^a^Dietary pattern scores were adjusted for energy intake by using the residual method.

^b^FFQ_R was administered 1 year after or before FFQ_V.

^c^FFQ_V was administered after completion of the DRs.

^d^28- or 14-day DRs were collected over a 1-year period.

All correlations: *P* < 0.001.

## DISCUSSION

In a subsample of the validation study of diet in the JPHC Study, we identified 3 major Japanese dietary patterns (prudent, westernized, and traditional) that were similar across data sources and between sexes. The reproducibility and validity of dietary patterns derived from the FFQ were acceptable. Spearman correlation coefficients between scores of the dietary patterns derived from the 2 FFQs ranged from 0.55 to 0.77. The corresponding values between scores of the dietary patterns derived from the dietary records and those derived from the FFQ ranged from 0.32 to 0.63. These dietary patterns were also identified in the entire population of the JPHC Study.

The 3 major dietary patterns identified in the present study have also been reported in previous Japanese studies. Most studies in Japan have identified a dietary pattern similar to the prudent Japanese pattern in the present study.^19^^–^^35^ This dietary pattern is characterized by high intakes of not only vegetables and fruit but also typical Japanese foods, including soy products, seaweed, mushrooms, fish, and green tea. Dietary patterns similar to the westernized Japanese pattern in the present study, ie, high intakes of meat, processed meat, bread, coffee, black tea, mayonnaise, and dressing, have also been observed in many Japanese studies.^19^^–^^35^ Moreover, the traditional Japanese pattern, which is characterized by high intakes of rice, miso soup, pickles, salmon, salty fish, and fruit, has been observed in several studies.^19^^–^^21^^,^^26^^,^^30^^,^^31^ Because the westernized breakfast pattern^22^^,^^24^^,^^28^^,^^29^^,^^33^^,^^34^ and similar patterns, which were identified in some other studies, is associated with low intakes of rice and miso soup, the westernized breakfast pattern could be viewed as the mirror image of the traditional Japanese pattern.

We observed acceptable validity and reproducibility of the 3 dietary patterns; the Spearman correlation coefficients between the dietary records and 2 FFQs ranged from 0.55 to 0.77 for reproducibility and from 0.32 to 0.63 for validity. These values were comparable to those reported in previous studies. Regarding reproducibility, the correlation coefficients ranged from 0.67 to 0.70 (Pearson) in the Health Professional Follow-up Study,^9^ from 0.63 to 0.73 (over a 1-year period; Spearman),^10^ and from 0.30 to 0.52 (over a 10-year period; Pearson)^11^ in the Swedish Mammography Cohort, and from 0.64 to 0.81 (Spearman) in the Southampton Women’s Survey.^7^ Regarding validity, the correlation coefficients ranged from 0.34 to 0.64 (Pearson) in the Health Professionals Follow-up Study,^9^ from 0.34 to 0.61 (Pearson) in the Monitoring of Trends and Determinants in Cardiovascular Diseases,^13^ from 0.41 to 0.73 (Spearman) in the Swedish Mammography Cohort,^10^ from 0.35 to 0.67 (Pearson) in a UK study,^8^ and from 0.36 to 0.62 (Pearson) in a Japanese study.^12^

In the present study, the traditional Japanese pattern in men and the westernized Japanese pattern in women were highly reproducible. In the 2 FFQs, salty fish, salmon, pickles, *sake*, and rice, which are characteristic of the traditional Japanese pattern in men, showed high loadings to the pattern. In addition, these food intakes were highly correlated between the 2 FFQs. Similarly, intakes of beef, processed meat, coffee, bread, pork, chicken, and dressing showed high loadings to the westernized Japanese pattern in women in the 2 FFQs and were also highly correlated between the 2 FFQs. Of the 3 dietary patterns identified, the traditional Japanese pattern showed relatively good validity in both men and women. This might be because pickles, rice, salty fish, salmon, and *sake* (men only) showed high factor loadings to both dietary record and FFQ, and these intakes were moderately to highly correlated between the dietary record and FFQ. In contrast, the westernized Japanese pattern in men was less valid, perhaps because among foods that were highly and positively loaded to the westernized Japanese pattern derived using dietary record data, some fish items were inversely (not positively) associated with the dietary pattern based on FFQ data. In addition, intakes of meat and processed meat were only moderately correlated between dietary record and FFQ.

Regarding the traditional Japanese pattern in women, the factor loadings from the FFQ in the subsample and those from the FFQ in the overall cohort for food groups were not highly correlated (correlation coefficient = 0.47). This might be because factor loadings of pickles, citrus fruit, and noodles in the traditional Japanese pattern were lower in the overall cohort than in the subsample, whereas those for pork, beef, processed meat, egg, oily fish, and fish products were higher in the overall cohort than in the subsample.

Our study had some limitations. First, although we assessed the validity and reproducibility of dietary patterns in a subsample of the JPHC Study population, participants in the validation study might be more health conscious than nonparticipants. Therefore, the present findings might not be applicable to the entire population of the JPHC Study. However, we also identified 3 similar dietary patterns in the entire population. Second, we observed similar dietary patterns in men and women. This might be partly because married couples were recruited for the validity study. Third, we assessed validity based on a single measurement of dietary intake, which might not reflect past dietary patterns. In addition, dietary pattern alone could change during the follow-up period, due to changes in food preference and food availability. Finally, we used the FFQ completed at the end of the dietary records to evaluate validity. Participant recall of dietary intake could be influenced by the administration of dietary records.

In conclusion, we identified 3 Japanese dietary patterns (prudent, westernized, and traditional) using FFQ data among participants of the validation study and confirmed that validity and reproducibility of the FFQ are acceptable. These validation data provide a basis for future analysis of the association between dietary patterns and disease risk in the JPHC Study population.

**Appendix.** Factor-loading matrix for major Japanese dietary patterns identified by principal component analysis^a^ in the JPHC study (*n* = 101 630)

	Men (*n* = 47 408)			Women (*n* = 54 222)			All (*n* = 101 630)		
			
	Prudent	Westernized	Traditional	Prudent	Westernized	Traditional	Prudent	Westernized	Traditional
Noodle	0.54	0.21		0.53	0.20	0.14	0.47	0.24	0.14
Rice			0.20			0.17			0.19
Bread	0.35	0.34	−0.17	0.43	0.35		0.37	0.40	−0.16
Potatoes	0.66	0.13		0.71			0.68	0.16	
Soy products	0.65			0.66			0.61	0.11	
Miso soup	0.16		0.17	0.12		0.20	0.13		0.21
Nuts	0.30	0.33	0.19	0.31	0.21	0.25	0.30	0.28	0.21
Green, leafy vegetables	0.70	0.10		0.64		0.19	0.65	0.13	0.11
Other green vegetables	0.65	0.19		0.64		0.25	0.65	0.18	0.12
Dark-yellow vegetables	0.63			0.53			0.55	0.12	
Tomatoes	0.44	0.24	0.17	0.45	0.13	0.25	0.46	0.21	0.17
Other vegetables	0.77			0.69		0.14	0.70		
Pickles	0.43		0.47	0.44	−0.19	0.39	0.51	−0.11	0.33
Other fruit	0.71		0.12	0.73		0.21	0.74		
Citrus fruit	0.57		0.16	0.61		0.19	0.64		
Fruit juice	0.14	0.42		0.16	0.31	0.12	0.15	0.39	
Mushroom	0.58	0.19	0.27	0.63	0.14	0.19	0.62	0.18	0.17
Seaweed	0.70			0.71			0.68	0.11	
Seafood other than fish	0.42	0.29	0.40	0.35	0.11	0.55	0.41	0.19	0.49
Oily fish	0.45	0.19	0.32	0.36		0.47	0.42	0.12	0.41
Salmon	0.18	0.22	0.62	0.18		0.61	0.27		0.53
Eel	0.12	0.44	0.12		0.38	0.25		0.39	0.28
Lean fish	0.30	0.30	0.31	0.23	0.18	0.49	0.26	0.22	0.46
Salty fish	0.21		0.72	0.24		0.59	0.34		0.53
Fish products	0.42	0.30		0.33	0.15	0.31	0.38	0.26	0.17
Pork	0.25	0.40	0.17		0.31	0.44	0.15	0.35	0.36
Beef		0.48	0.12		0.43	0.30		0.44	0.30
Chicken	0.15	0.41	0.35		0.26	0.56	0.13	0.32	0.46
Liver		0.38	0.26		0.28	0.37		0.30	0.39
Processed meats	0.20	0.51		0.12	0.45	0.38	0.15	0.50	0.25
Eggs	0.30	0.19	0.16	0.21	0.15	0.33	0.24	0.18	0.27
Milk	0.21	0.17		0.16	0.20	0.20	0.22	0.21	
Dairy products	0.23	0.45		0.24	0.35	0.26	0.29	0.42	
Soup		0.55	−0.15	0.14	0.57			0.58	
Confectionaries	0.37	0.35		0.47	0.30	0.20	0.46	0.37	
Green tea	0.34		0.17	0.38			0.37		
Coffee		0.39		0.15	0.48			0.45	
Soft drink		0.43			0.35			0.40	
Oolong tea		0.48	−0.16	0.13	0.46			0.49	
Black tea		0.48	−0.10	0.16	0.51		0.11	0.51	−0.10
Sauce	0.34	0.47		0.39	0.46	0.14	0.35	0.49	
Mayonnaise	0.33	0.43		0.39	0.38	0.18	0.37	0.43	
Dressing	0.26	0.52		0.32	0.53	0.12	0.29	0.55	
Sake			0.53			0.11		−0.12	0.46
Shochu			−0.32		0.14		−0.20		0.13
Beer		0.30			0.28		−0.18	0.21	0.33
Whisky		0.17			0.16		−0.12	0.11	0.20
Wine		0.16			0.24			0.16	

Abbreviation: JPHC Study, Japan Public Health Center-Based Prospective Study.

^a^For simplicity, factor loadings less than ±0.10 are not listed.
